# Transmission of COVID-19 From an Asymptomatic Mother to Fetus Leading to Death of Neonate From Pneumonia

**DOI:** 10.7759/cureus.21999

**Published:** 2022-02-07

**Authors:** Anthony Sanchez, Maryellen Campbell, Sai Palati, Martin Castaneda

**Affiliations:** 1 College of Medicine, Florida Atlantic University Charles E. Schmidt College of Medicine, Boca Raton, USA; 2 Obstetrics and Gynecology, Bethesda Hospital East, Boynton Beach, USA

**Keywords:** remdesivir, neonatal respiratory distress, neonatal intensive care management, early neonatal deaths, transmission in neonatal covid-19, covid-19 pneumonia, new borns, anti viral drugs, vertical infectious disease transmission, covid 19

## Abstract

The severe acute respiratory syndrome coronavirus 2 (SARS-CoV-2) responsible for the coronavirus disease 2019 (COVID-19) pandemic has rarely impacted neonates. When infection does occur, it is typically asymptomatic. We describe a case of a neonate born to a 25-year-old mother who was COVID-19 positive but asymptomatic. An emergent cesarean section was performed during week 30 of gestation due to category three fetal heart tracings. The neonate, unfortunately, died on the day of life 12 from respiratory distress secondary to severe COVID-19 pneumonia. This is an important case that illustrates the deleterious impact COVID-19 infection can have on neonates. It is a unique case of the compassionate use of remdesivir for a neonate. The patient's respiratory decline soon after birth, lends support that the virus responsible for COVID-19 can be transmitted vertically.

## Introduction

Pneumonia is a significant cause of morbidity and mortality in premature infants. Infants can acquire pneumonia through many different routes including transplacentally, via infected amniotic fluid, via colonization at delivery, or nosocomially [1]. Maternal infection, prematurity, and prolonged rupture of membranes are risk factors for developing neonatal pneumonia [1]. Newborns are at an elevated risk of infection due to their immature immune systems with diminished cellular and humoral function. They also have underdeveloped respiratory cilia and fewer pulmonary macrophages. Infants presenting with signs such as apnea, bradycardia, cyanosis, lethargy, and increased respiratory effort must be evaluated for sepsis with blood culture and cerebral spinal fluid (CSF) culture. Broad-spectrum antibiotics should be initiated as early as possible [2].

The severe acute respiratory syndrome coronavirus 2 (SARS-CoV-2) virus responsible for the global pandemic causes a spectrum of disease manifestations ranging from asymptomatic to severe acute respiratory syndrome and death. While pediatric patients infected with SARS-CoV-2 have typically not had severe symptoms and are rarely affected, they are a particularly vulnerable population due to their still developing immune systems [3].

Few studies have shown the effects of maternal COVID-19 infection on vertical transmission in utero and perinatal transmission to neonates. A case report published in the Italian Journal of Pediatrics described two neonates born from mothers with COVID-19 pneumonia that subsequently developed COVID-19 infection. They presented with lymphopenia, elevated LDH, and hypocalcemia immediately after birth [1]. Notably, their first reverse transcriptase-polymerase chain reaction (RT-PCR) tests for COVID-19 were negative, and their RT-PCR was not positive for COVID-19 until days 7 and 12 of life, respectively. Both patients were treated with hydroxychloroquine and recovered.

## Case presentation

This is a case of a 12-day-old infant born to an asymptomatic COVID-19 positive G1P0 mother at gestational age 30 weeks and four days by emergent cesarean section (c-section) who died of a severe case of COVID-19 pneumonia.

The mother is a 25-year-old primigravid with a past medical history of a polycystic ovarian syndrome (PCOS). She tested positive for COVID-19 via RT-PCR during hospital admission for labor, but she was asymptomatic. She was negative for group B streptococcus, HIV, and hepatitis B. She had no known allergies and did not smoke or use alcohol or other substances during her pregnancy. She had no history of gestational diabetes. Her only medication was Bonjesta for gravid nausea. At presentation, the fetal heart rate tracing was found to be category III and she was taken to the operating room (OR) for an emergency c-section. She received ampicillin, magnesium sulfate, steroids, IV fluids, and oxygen prior to the emergency c-section. No maternal sedation was given. The mother’s chest x-ray (CXR) after the c-section did not reveal consolidations. 

**Figure 1:**
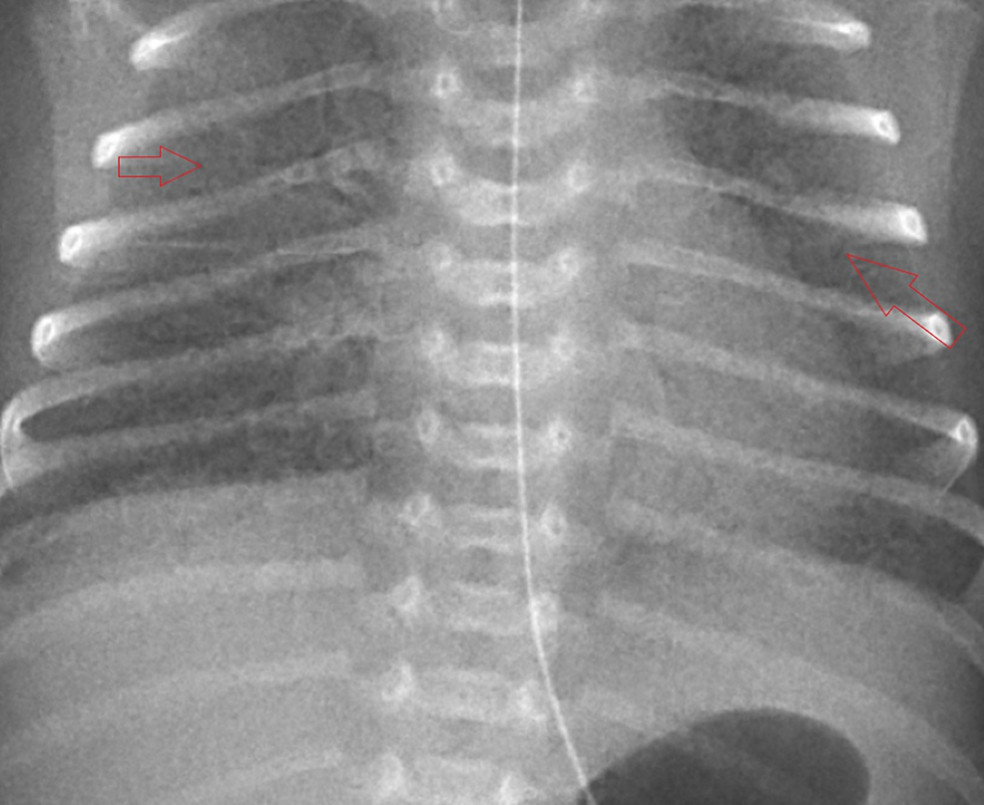
Chest x-ray taken immediately after birth indicate bilateral interstitial infiltrates (arrows)

C-section was performed due to category III tracings from fetal decelerations. Appearance Pulse Grimace Activity Respiration (APGAR) scores were six and seven at one and five minutes, respectively. The patient’s birth weight was 1,300 grams. There was no meconium in the amniotic fluid. The patient was intubated immediately in the delivery room due to respiratory distress syndrome type one. After intubation, heart rate was 165 beats per minute, respirations were 38 breaths per minute, and oxygen (O_2_) saturation was 92%. An RT-PCR sample was obtained from the patient in the OR after intubation. A specimen for aerobic blood culture was also collected at this time. Ampicillin and gentamicin antibiotic therapy was started for suspected early-onset sepsis management following blood culture collection. Synchronized intermittent mandatory ventilation with pressure support (SIMV-PS) was initiated. Portable CXR displayed moderate interstitial lung disease (Figure 1), and arterial blood gas (ABG) showed pH of 7.47, partial pressure of carbon dioxide (pCO_2_) of 27 millimeters of mercury (mmHg), partial pressure of oxygen (pO_2_) of 31 mmHg, bicarbonate (HCO_3_) of 19.7 millimoles per liter (mmol/L) (see Table 1 for ABG values from all days). The patient did not have any considerable time with the mother in the delivery room. Amniotic/placental samples were not collected. No anatomical abnormalities were noted on ultrasound at this time. The patient was taken to the neonatal intensive care unit (NICU) following intubation, where IV fluids and total parenteral nutrition (TPN) were initiated.

**Table 1:** Arterial blood gas values for each day of life pCO_2_ = partial pressure of carbon dioxide, pO_2_ = partial pressure of oxygen, HCO_3_ = bicarbonate

ABG Parameter	Normal	Day 1	Day 2	Day 3	Day 4	Day 5	Day 6	Day 7	Day 8	Day 9	Day 10	Day 11	Day 12
pH	7.32 - 7.43	7.47	7.37	7.26	7.15	7.4	7.35	7.46	7.31	7.37	7.32	7.27	7.3
pCO_2_ (mmHg)	35 - 45	27	35	49	44	40	38	41	53	45	47	54	52
pO_2_ (mmHg)	75 - 100	31	69	37	45	50	48	50	129	49	37	74	31
HCO_3_ (mmol/L)	22 - 26	19.7	20.2	22	15.3	24.8	21	29.2	26.7	26	24.2	24.8	25.6

**Figure 2:**
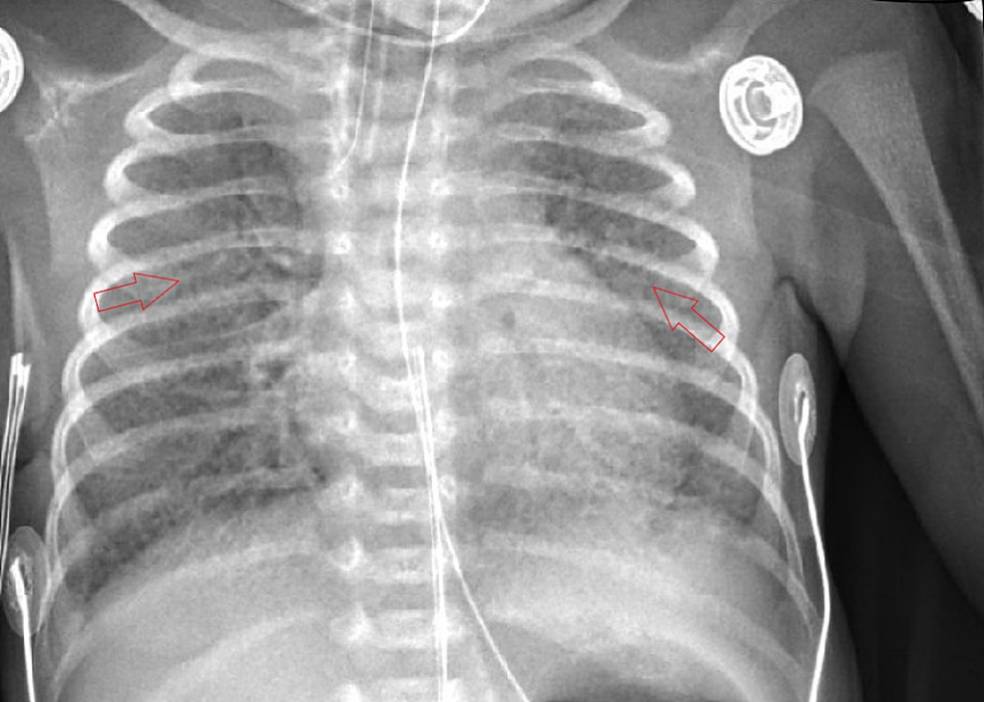
CXR from day 2 of life show diffuse, bilateral, reticulogranular opacification (arrows) CXR - chest x-ray

On day 2 of life, lung imaging showed diffuse, bilateral, reticulogranular opacification (Figure 2). RT-PCR returned positive for COVID-19. Surfactant was administered (poractant alfa, 1.79mg).

On day 3, lung imaging remained unchanged. The patient was started on dexamethasone at a total of 0.15 mg/kg/day divided into two doses per day.

On day 4, CXR showed no interval change in bilateral lung aeration. The patient's clinical course remained unchanged this day.

On day 5, remdesivir was considered but not started because it is not approved for use in newborns under 3.5 kg. Coarse transmitted lung sounds were noted along with a systolic heart murmur. Aerobic blood cultures showed no growth after five days. CXR displayed unchanged persistence of bilateral infiltrates.

**Figure 3:**
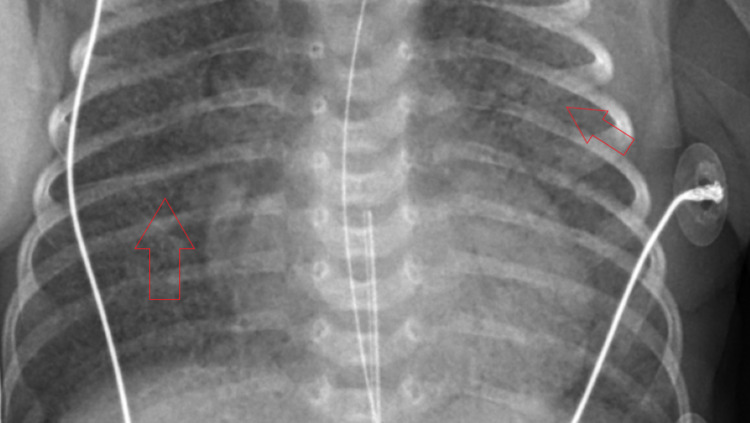
CXR on day 6 of life display granular infiltrates, consistent with hyaline membrane disease (arrows) CXR - chest x-ray

**Figure 4:**
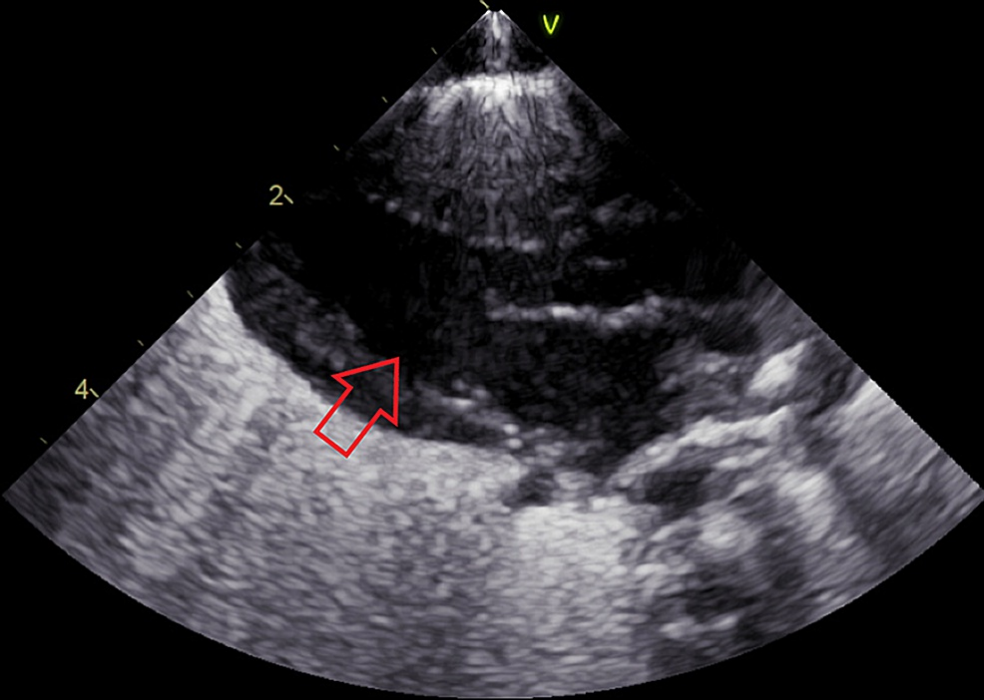
Echocardiogram on day 6 of life shows large patent foramen ovale (arrow)

On day 6, imaging revealed granular infiltrates consistent with hyaline membrane disease (Figure 3). The patient developed mild thrombocytopenia with a platelet count of 139,000 platelets/mcL. Hematology was consulted for guidance concerning how to manage anticoagulation in the setting of COVID-19 infection hypercoagulability. It was determined that anticoagulation should be avoided due to the lack of NICU guidelines for treatment, degree of prematurity, and risk of bleeding from the patient’s thrombocytopenia. Hematology recommended d-dimer monitoring and initiation of anticoagulation only if a thrombus was diagnosed. The manufacturer of remdesivir was contacted to determine if the patient met the criteria for inclusion into any current trials, but she did not. An echocardiogram (Figure 4) showed a large patent foramen ovale (PFO), patent ductus arteriosus (PDA) with a right to left shunt, and mild tricuspid regurgitation. Systolic pulmonary pressures were noted to be 64 mmHg above systemic circulation. The troponin level was 14 ng/mL.

On day 7, the patient decompensated due to an acute exacerbation of respiratory distress. The use of remdesivir was discussed with the mother, highlighting the fact that little data is available to determine impacts on a baby this age and size. Potential side effects were also discussed such as transaminitis, and the mother agreed to begin remdesivir. The patient was started on remdesivir at a loading dose of 2.5 mg/kg and 1.25 mg/kg/day for each subsequent day for five days. Infusion of remdesivir was well-tolerated. Troponin was elevated from the previous day at 18 ng/mL.

**Figure 5:**
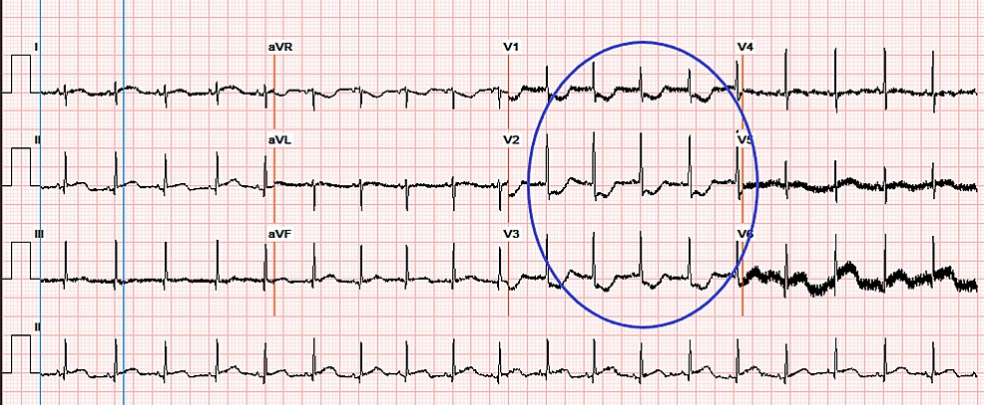
EKG on day 8 of life indicates leads showing ST depressions (circle)

On day 8, an electrocardiogram (EKG) demonstrated ST depressions (Figure 5), and troponin was noted to be further elevated at 22 ng/mL. D-dimer was 19 mcg/mL.

**Figure 6:**
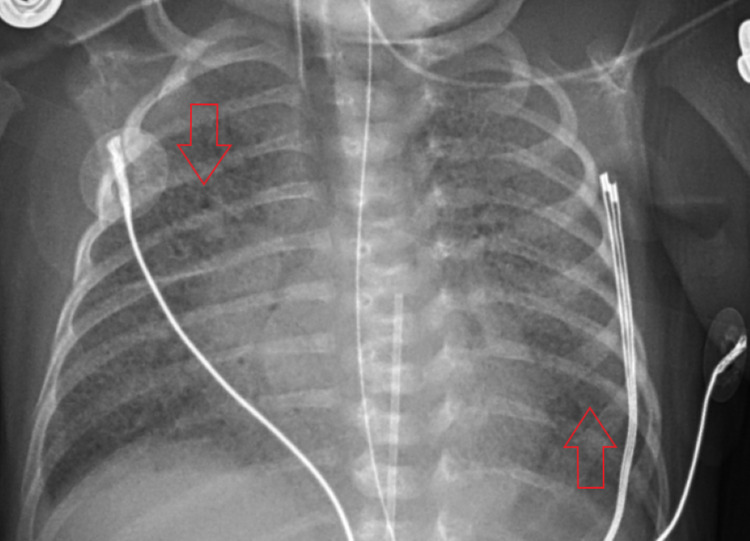
CXR from day 9 of life shows worsening of diffuse, bilateral granular infiltrates (arrows) CXR -  chest x-ray

On day 9, large, diffuse, bilateral granular infiltrates were found on imaging (Figure 6). Troponin decreased to 13 ng/mL. A repeat echocardiogram showed unchanged PFO with reversal of PDA shunt, now left to right. Polymerase chain reaction (PCR) testing was positive for COVID-19 antibodies.

On day 10, bilateral crackles were noted on the exam. CXR showed unchanged persistence of interstitial infiltrates.

On day 11, the last dose of remdesivir was administered. The patient's clinical course remained unchanged.

**Figure 7:**
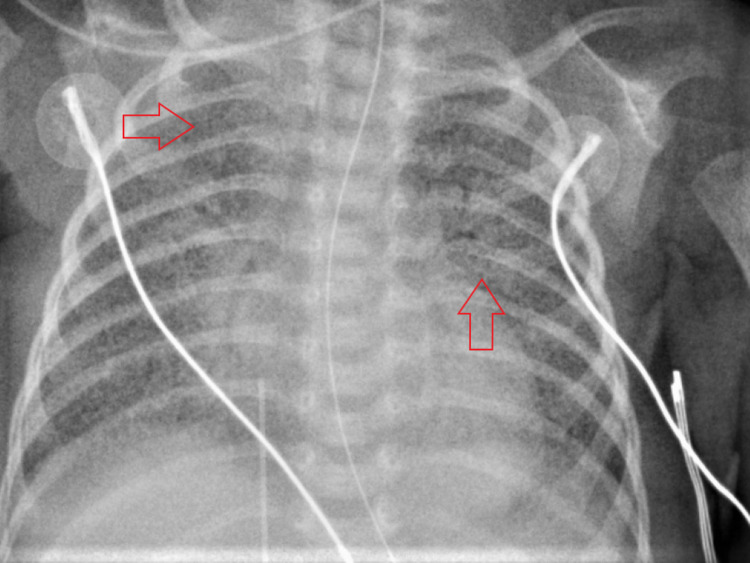
CXR from the last day of life show coarse, bilateral, parenchymal infiltrates (arrows) CXR - chest x-ray

**Figure 8:**
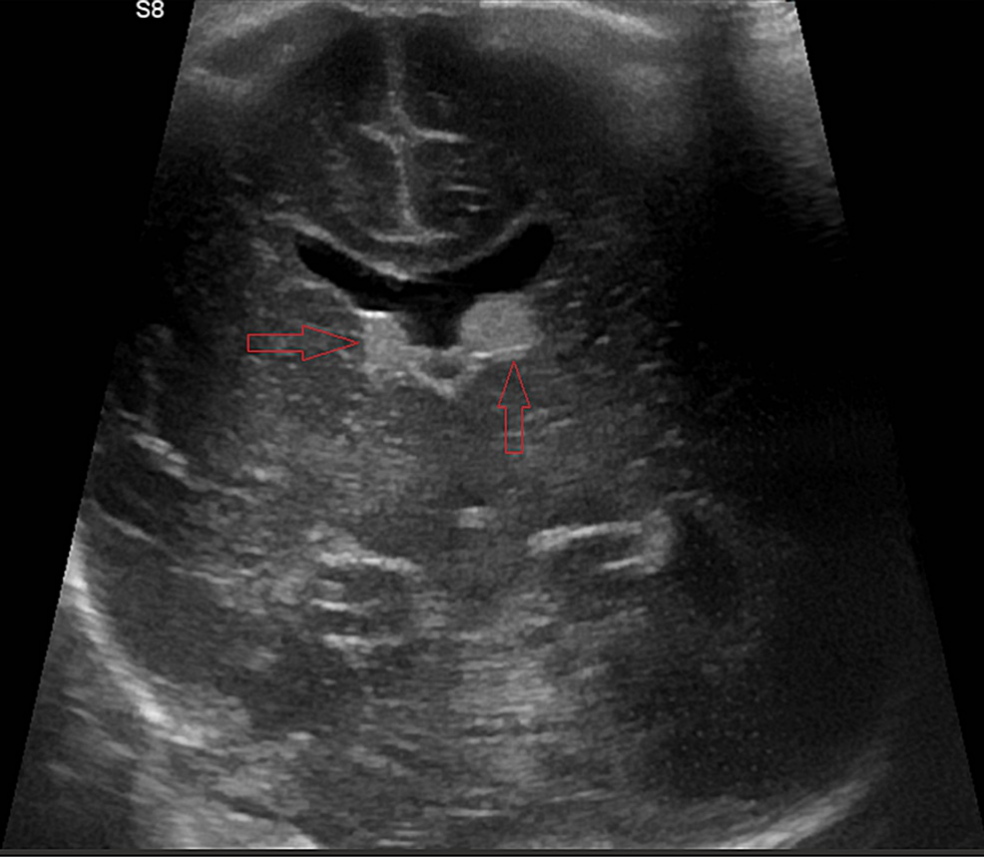
Neurosonography from the final day of life displays bilateral hematomas in the caudothalamic grooves (arrows)

On day 12, coarse bilateral parenchymal infiltrates were noted on imaging (Figure 7). An audible air leak was appreciated on an exam. The patient developed a low total hemoglobin mass (THB) of 9.1 grams per deciliter (g/dL). Neurosonography displayed interval development of echogenic material at the caudothalamic grooves bilaterally, consistent with hematomas (Figure 8). The left hematoma measured 5 mm and the right measured 4 mm. The patient expired the next day. The cause of death was respiratory distress secondary to severe COVID-19 pneumonia.

## Discussion

This case highlights the importance of further investigation into the effects of COVID-19 on fetuses and neonates. Current research shows that there are three potential mechanisms through which neonates can become infected with COVID-19: 1) vertical transmission through transplacental hematogenous spread or viral particles in amniotic fluid, 2) intrapartum transmission after exposure to infected maternal secretions or feces, and 3) postpartum transmission from an infected mother or other close contacts including healthcare personnel [4]. Initially, it was believed that vertical transmission of COVID-19 was not possible; however, new data are showing that while the risk is low, there is still a possibility of vertical transmission [5]. Due to the lack of placental or amniotic fluid samples, in this case, it is impossible to definitively say the patient was infected via vertical transmission as opposed to a respiratory transmission from aerosolized viral particles. However, the patient's positive RT-PCR taken at delivery along with the extent of pulmonary damage found on the first CXR taken shortly after delivery are suggestive of vertical transmission and infection.

Current studies show that most neonates infected with COVID-19 are asymptomatic or have mild symptoms including rhinorrhea and cough [4]. However, compared to older children, neonates tend to have higher rates of severe illness that require ICU care [4]. If symptomatic, most neonates present with any potential combination of cough, fever, rhinorrhea, respiratory distress, poor feeding, lethargy, vomiting, diarrhea, rash, and edema [4]. Preterm infants with early-onset sepsis, whose mothers were infected with COVID-19, typically present with apnea, bradycardia, cyanosis, lethargy, and increased respiratory effort [3].

In the pediatric population, COVID-19 infection has rarely been associated with cardiovascular complications including arrhythmias, myocarditis, heart failure, and myocardial infarction [6]. This is a possible explanation for the transient ST depressions on EKG discovered in this patient. Another important sequela of COVID-19 is multisystem inflammatory syndrome in children (MISC). The presentation includes a wide variety of symptoms involving cardiovascular, gastrointestinal symptoms, and mucocutaneous symptoms resembling Kawasaki's disease. Pathologic sequela includes elevated cardiac enzymes, shock, coronary artery abnormalities, neurologic manifestations, nausea, vomiting, and diarrhea mimicking gastroenteritis [4].

Due to the paucity of research on COVID-19 infection in neonates, there are currently no guidelines for management and treatment in this population. Most guidelines focus on the care of neonates born to COVID-19 positive mothers to prevent infection but do not address treatment measures if infected [7]. Therefore, protocols for adult treatment are followed with modifications for this cohort of patients [8]. Most treatment options focus on providing supportive care for the neonate including fluid and electrolyte support. For respiratory failure, nasal continuous positive airway pressure should be given and intubation with mechanical ventilation should be initiated for severe respiratory failure. Other treatment options in severe respiratory failure include inhaled nitric oxide, surfactant, and high-frequency oscillatory ventilation. Antiviral and antibiotic drugs should be considered on a case-by-case basis [9]. The data regarding the efficacy of remdesivir in neonates is extremely limited [10,11]. In those cases, and the present case, remdesivir was administered as compassionate therapy due to the deteriorating clinical picture.

## Conclusions

This case emphasizes the need for further research and discussion on the effects of COVID-19 on neonates along with more research concerning the vertical transmission of the SARS-CoV-2 virus. Adequate guidelines for the management of COVID-19 infection in this population are lacking due to limited data. The effects of COVID-19 infection on neonates may also be more severe than current scientific literature seems to suggest. This case illustrates how significant morbidity and mortality can result from COVID-19 infection in neonates and can result in rapid deterioration and demise. The importance of vaccination against COVID-19 for pregnant mothers to reduce the risk for themselves and their children should not be underestimated.
